# Prevalence and Factors Associated With the Risk of Delayed Sleep-Wake Phase Disorder in Japanese Youth

**DOI:** 10.3389/fpsyt.2022.878042

**Published:** 2022-05-13

**Authors:** Sayaka Tomishima, Yoko Komada, Kosuke Tanioka, Isa Okajima, Yuichi Inoue

**Affiliations:** ^1^Department of Somnology, Tokyo Medical University, Tokyo, Japan; ^2^Faculty of Liberal Arts, Meiji Pharmaceutical University, Tokyo, Japan; ^3^Japan Somnology Center, Institute of Neuropsychiatry, Tokyo, Japan; ^4^Department of Psychological Counseling, Faculty of Humanities, Tokyo Kasei University, Tokyo, Japan

**Keywords:** delayed sleep-wake phase disorder, biological rhythms interview of assessment in neuropsychiatry (BRIAN), web-based questionnaire, young generation, daytime function

## Abstract

**Background:**

Delayed sleep-wake phase disorder (DSWPD) is more prevalent among the younger generation. However, the prevalence of this disorder in Asia, particularly Japan, has not yet been elucidated. Furthermore, the impact of DSWPD morbidity on daytime functioning and factors associated with the presence of the disorder remain unclear.

**Methods:**

A web-based survey was conducted among youth aged 15–30 years. In total, 7,810 individuals completed the questionnaire. The questionnaire included items on sociodemographic variables as well as the Japanese version of the Biological Rhythms Interview of Assessment in Neuropsychiatry self-report (J-BRIAN-SR), which assesses the risk of DSWPD, sleep behaviors and possibly related lifestyle variables, productivity loss [WHO Health and Work Performance Questionnaire (HPQ)], and health-related quality of life (HRQOL). The risk of DSWPD was indicated by a J-BRIAN-SR score greater than or equal to 40 points and days of absence ≥4 days per month. After comparing these variables for participants at risk of DSWPD and those who were not, the factors associated with the risk of DSWPD were examined using logistic regression analysis, with sociodemographic and lifestyle variables as independent variables.

**Results:**

The overall prevalence of participants at risk DSWPDs was 4.3%. Compared with participants without DSWPD, those at risk of DSWPD presented significantly worse HPQ and HRQOL scores. The risk of DSWPD was positively associated with the presence of currently treated diseases, length of nighttime liquid crystal display (LCD) viewing, and being a high school/university students. It was negatively associated with habitual exercise.

**Conclusion:**

The risk of DSWPD seemed to be consistent with reports from Western countries, and individuals possibly affected by the disorder were thought to have deteriorated daytime functioning. In addition, lifestyle specific to youth, such as long-term LCD viewing at night and relatively loose social constraints, could be associated with the presence of DSWPD in this generation.

## Introduction

Delayed sleep-wake phase disorder (DSWPD) is a circadian rhythm disorder characterized by the delay of major sleep episodes relative to their clock time, resulting in sleep-onset insomnia and difficulty waking up at the desired time in the morning with concomitant daytime dysfunction for at least 3 months (1–3). This disorder is more prevalent among young generation, in which a delay in circadian phase preference has been identified (4, 5). The prevalence rate of DSWPD has been reported at 0.17%−1.5% in the general population (6, 7). In comparison, a survey on 10,220 adolescents aged 16–18 years in Norway found a relatively higher rate of 3.3% (8), and a most recent Norwegian survey of 50,054 students aged 18–35 years showed a prevalence of 3.3% (9), suggesting that the disorder is possibly more prevalent in the younger generation than in other populations. The desired bed-off time of workers in their 20s is much earlier than that of university students (10), and the desynchronization of their sleep phase due to DSWPD may lead to maladaptation to their work life (11). However, in Japan, there has not been a real-world epidemiological survey of younger generation targeting both students and workers. Moreover, although information relating to an individual's desired bedtime is required for DSWPD diagnosis, this information was not included in the self-reported questionnaire employed in previous epidemiological studies (9, 12, 13). Some studies have reported that adolescents with DSWPD have a higher prevalence of mental health problems such as depression and anxiety (3, 13, 14). However, to date, no studies have comprehensively investigated the overall impact of DSWPD on daytime function and social function, including presenteeism and absenteeism. Although the pathogenesis of DSWPD has been suggested to be partially dependent on genetic factors (15), the detailed mechanism of the development of the disorder has not yet been clarified. Considering these issues, this study aimed (1) to clarify the prevalence of individuals at risk of DSWPD among the young Japanese population using J-BRIAN-SR, which has already been validated for DSWPD screening (16), (2) to investigate the impact of the risk of DSWPD on daytime function, and (3) to examine sociodemographic and lifestyle-related factors of the risk of DSWPD.

## Methods

### Study Population

The study protocol was approved by the Ethics Committee of the Neuropsychiatric Research Institute (Tokyo, Japan). This web-based, cross-sectional study recruited participants *via* an online marketing research company (Macromill.inc.) with a cohort of approximately 10 million Japanese. The survey was conducted in October 2019. An e-mail containing a link to an online questionnaire was randomly sent by the research company to individuals throughout Japan who were stratified by district, sex, and age. The participants' ages ranged from 15 to 30 years old. Informed consent was obtained from all participants before they responded to the questionnaire. A total of 10,303 completed questionnaires were received. Of these, the following were excluded from subsequent analyses: (1) participants who provided obvious invalid responses (e.g., gave answers that were inconsistent with those given for other questions or figures that exceeded standard deviations of 3.0; *n* = 1,582); (2) shift workers (e.g., rotating shifts, and night or early morning shifts; *n* = 911). As a result, data from 7,810 participants were included in subsequent analyses.

### Measures

#### Sociodemographic Characteristics

Participants provided answers to items relating to their sex, age, height, weight, occupation (regular worker, part-time worker, student, and unemployed), family constitution (Do you currently live alone or with your family?, and do you have any children?), a habit of physical exercise [Do you have a habit of physical exercise (more than 2 days a week, more than 30 min a day, for a duration longer than a year)?], presence/absence of currently treated diseases (Are you currently receiving treatment for any diseases?), monitor viewing time [How long do you watch liquid crystal displays (LCDs) such as TV, computer, mobile, etc. after 6 pm and before you go to sleep?]; time spent outdoors on weekdays and weekends [How much time do you spend outdoors (without an overhead roof) during the day?], commuting time, and working hours. The participants were also required to report the number of days in the previous month with a poor physical condition that caused them to miss school or work or left them unable to perform daily routines. In addition, only those aged 20 years or older were asked about their smoking status (Do you currently smoke?) and habitual alcohol ingestion (Do you habitually drink alcohol?). Body mass index (BMI) was calculated based on the self-reported body weight (kg) divided by the squared height (m^2^).

#### Subjective Sleep Variables

Habitual bedtime (What time do you usually go to bed on weekdays?), wake-up time (What time do you usually get up on weekdays?), and sleep onset latency (SOL; How long does it usually take you to fall asleep?) with regard to the participants' sleep habits, were assessed separately for workdays and free days, respectively. Time in bed (TIB) was calculated based on the difference between bedtime and wake-up time. Sleep onset was defined as bedtime plus sleep onset latency, and sleep duration was defined as wake-up time minus sleep onset. We calculated the social jet lag (SJL) based on the difference between the mid-sleep on work- and free days. Mid-Sleep Time on free days was corrected for sleep debt on free days (MSFsc) (17, 18).

#### Screening of Delayed Sleep-Wake Phase Disorder

To screen individuals at risk of DSWPD, we employed the following criteria: (1) having 40 or more points in the Japanese version of the Biological Rhythms Interview of Assessment in Neuropsychiatry Self Report (J-BRIAN-SR) total score as reported by Kanda et al. (16) and (2) missing work, school or daily routines for 4 or more days per month. An absence of 4 or more days per month, based on a report showing that school absence due to DSWPD may become a factor causing poor academic performance (19), can be considered a conventional indicator of absenteeism according to surveys conducted in Japan (20).

#### Daytime Sleepiness

The Japanese version of the Epworth Sleepiness Scale (JESS), a measure of subjective daytime sleepiness, consists of eight items on the likelihood of falling asleep in sedentary situations (21, 22). Response options ranged from 0 (would never doze) to 3 (high chance of dozing). The total score was used to categorize respondents into those with “normal” (JESS <11) and those having “excessive daytime sleepiness” (JESS ≥11).

#### Insomnia

The Japanese version of the Athens Insomnia Scale (AIS-J) is a self-rated inventory consisting of eight items (23). Each item is rated from 0 to 3 with a total score ranging from 0 to 24 (24). The total cut-off score in the original AIS for identifying pathological insomnia was set at six points (25). We defined insomnia as AIS-J ≥6, according to a study on the validity and diagnostic utility of the scale (23).

#### Depression and Anxiety

The severity of depression symptoms was assessed using the Japanese version of the Kessler Psychological Distress Scale (K6), a screening tool with a 6-item questionnaire designed to detect symptoms of depression and anxiety (26). Response options ranged from 0 (never) to 5 (always). The total K6 score was used to categorize respondents into “without depression” (K6 <10) or “having depression” (K6 ≥10).

#### Health-Related Quality of Life

The health-related QOL of the participants was investigated using the Japanese version of the 8-item health-related quality of life survey (SF-8), which is a questionnaire for self-rated health and consists of eight items: physical function, physical roles (limitation of roles due to physical problems), bodily pain, general health, vitality, social function, emotional roles (limitation of roles due to emotional problems), and mental health (27). Physical component summary (PCS) and mental component summary (MCS) were calculated based on the factor structure of each subscale. PCS and MCS scores of <50 indicate poor physical and mental health, respectively (28).

#### Presenteeism

Presenteeism was measured using the Japanese version of the Short-Form Work Limitations Questionnaire (WLQ-SF), a self-administered questionnaire that measures the negative effect of health problems on work performance (this is so-called “on-the-job disability” or “presenteeism”) (29–31). The WLQ-SF is an 8-item version derived from the original 25-items WLQ and was developed to provide a reliable and effective measurement tool in a more compact format. The WLQ productivity loss score, generated from WLQ-SF, estimates the rate of reduction in work productivity due to health problems over 2 weeks (29). The validity and reliability of the Japanese version of the WLQ productivity loss score have already been verified (31). Positivity for work limitations is defined when an individual has an at-work productivity loss score ≥5% on the Work Limitations Questionnaire (WLQ) (29, 32).

### Statistics

To examine differences in sociodemographic characteristics, sleep variables, and daytime dysfunction in participants at risk of DSWPD and those who were not*, t*-tests for quantitative data and chi-square tests for qualitative data were conducted together with calculation of between-group effect sizes using Cohen's *d*-formula and ϕ, respectively. Differences were considered significant when a *P*-value was <0.05. Typically, Cohen's *d*-values of 0.2 or below reflect a small effect size, around 0.50 reflect a moderate effect size, and 0.80 and above reflect a large effect size (33). Φ values of 0.1 or below also reflect a small effect size, around 0.3 reflect a moderate effect size, and 0.5 and above, which reflects a large effect size.

Univariate logistic regression analyses were initially performed using “at risk” of DSWPD/non-DSWPD as the dependent variable and variables chosen based on significant group differences in the *t*-test or chi-square tests as independent variables. Finally, multiple logistic regression analyses were conducted for all variables that showed significant correlations in the univariate models to test which variables were independently associated with the risk of DSWPD. Results from the logistic regression analysis were presented as odds ratios (ORs) and 95% confidence intervals (CIs), and variables were determined to be significant when the CI did not include the number 1.00. The statistical tests of ORs were based on the Wald statistics. These analyses did not include the number of days of missing work, school, or daily routine.

After a series of analyses of the overall participants, additional analyses were conducted on students and non-students. All statistical analyses were conducted using the SAS Enterprise Guide for Windows (version 7.1; SAS Institute, Inc., Cary, NC, USA).

## Results

### Demographic and Descriptive Variables of the Participants

The mean age of the 7,810 participants was 21.3 (SD 4.3) years, and the sample included a larger number of women (76.4%) than men (23.6%, *d* = 0.05, *P* < 0.001). Approximately 60% of participants were students. Table 1 shows the demographic and descriptive variables of all the analyzed participants. The majority were single and had no children. Those who had physical exercise habits and were currently receiving medications accounted for 32.6 and 12.0%, respectively. The participants spent 3.9 (SD 2.1) hours on LCD viewing after 18:00 until bedtime. Self-reported days per month away from school, work, or daily routine were 0.9 (SD 3.5) days, and working hours per day were 8.2 (SD 2.3) hours.

**Table 1:** Descriptive variables of the studied population (*n* = 7,810).

	**All (*****n*** **= 7,810)**			**Non-DSWPD (*****n*** **= 7,473)**			**At risk of DSWPD (*****n*** **= 337)**			***P*** **-Value**	**Effect size**
	***N***	**% /mean**	**SD**	***N***	**% /mean**	**SD**	***N***	**% /mean**	**SD**		**(*d* or ϕ)**
**Age**, ***n*****, mean, years**	7,810	21.3	4.3	7,473	21.3	4.3	337	20.0	3.8	<0.001	0.30
**Sex**, ***n*****, %**											
Female	5,967	76.4		5,676	76.0		291	86.4		<0.001	0.05
Male	1,843	23.6		1,797	24.0		46	13.7			
**Social status**, ***n*****, %**											
Regular worker	1,781	22.8		1,746	23.4		35	10.4		<0.001	0.10
Part-time worker or inoccupation	983	12.6		932	12.5		51	15.1			
Homemaker	553	7.1		545	7.3		8	2.4			
High school student	1,929	24.7		1,808	24.2		121	35.9			
Graduate school/ University/ College student	2,219	28.4		2,128	28.5		91	27.0			
Other student	345	4.4		314	4.2		31	9.2			
Having children, *n*, %	905	11.6		885	11.8		20	5.9		<0.001	0.04
Single, *n*, %	6,560	84.0		6,245	83.6		315	93.5		<0.001	0.05
Living alone, *n*, %	1,502	19.2		1,454	19.5		48	14.2		<0.05	0.03
Smoking habits, *n*, %	401	9.2		382	9.1		19	12.3		0.1863	0.02
Habitual alcohol ingestion, *n*, %	1,553	35.8		1,488	35.6		65	41.9		0.1037	0.02
Habitual exercise habit, *n*, %	2,544	32.6		2,452	32.8		92	27.3		<0.05	0.02
Disease currently treated, *n*, %	937	12.0		820	11.0		117	34.7		<0.001	0.15
Body mass index, *n*, mean, kg/m^2^	7,810	20.5	2.8	7,473	20.5	2.8	337	20.6	3.2	0.2525	0.04
Length of LCD viewing time, *n*, mean, h/day	7,810	3.9	2.1	7,473	3.9	2.1	337	5.0	2.8	<0.001	0.52
Length of sunlight exposure time on weekdays, *n*, mean, h/day	7,810	1.8	2.4	7,473	1.8	2.3	337	1.9	2.8	0.1781	0.04
Length of sunlight exposure time on weekends, *n*, mean, h/day	7,810	1.7	2.5	7,473	1.7	2.5	337	1.8	3.1	0.7168	0.04
Missed school/ work or daily routine, *n*, mean, days/month	7,810	0.9	3.5	7,473	0.5	2.0	337	11.1	9.5	<0.001	3.82
Working/ School h, *n*, mean, h/day	6,775	8.2	2.3	6,490	8.2	2.3	285	7.9	2.9	<0.05	0.13
Commuting time, *n*, mean, min/day	6,775	39.8	30.9	6,490	39.7	30.8	285	41.5	31.1	0.3399	0.06

*n, number; SD, standard deviation; LCD, liquid crystal display*.

*P-values and effect sizes were derived from the t-test and chi-square test analyses*.

### Sociodemographic Characteristics and the Risk of DSWPD

Among 7,810 participants, 337 met our criteria for the risk of DSWPD, and the risk of DSWPD was 4.3%, which was higher in women than in men (4.9% vs. 2.5%, *P* < 0.001). As shown in Table 1, there were significant differences in LCD viewing time with longer time in the group at risk of DSWPD than in the non-DSWPD group (5.0 h vs. 3.9 h, *P* < 0.001). Participants at risk of DSWPD showed a smaller proportion of individuals with children (5.9% vs. 11.8%; χ^2^ = 11.0; *P* < 0.001) and those who lived alone (14.2% vs. 19.5%; χ^2^ = 5.6; *P* = 0.0175). The group at risk of DSWPD included fewer participants who habitually exercised (27.3% vs. 32.8%; χ^2^ = 4.5; *P* = 0.0347). A larger proportion of participants at risk of DSWPD had a currently treated disease (34.7% vs. 11.0%; χ^2^ = 172.2; *P* < 0.001) and were more likely to be single (93.5% vs. 83.6%; χ^2^ = 23.5; *P* < 0.001). Furthermore, a greater difference was observed in social status composition (χ^2^ = 73.0, *P* < 0.001); the proportion of students among the participants at risk of DSWPD was higher than that among the non-DSWPD group (72.1% vs. 56.9%; χ^2^ = 30.6; *P* < 0.001). However, there were no significant differences between the participants at risk of DSWPD and the non-DSWPD group in terms of the number of subjects with smoking habits (χ^2^ = 1.7, *P* = 0.1863), drinking habits (χ^2^ = 2.6, *P* = 0.1037), BMI (*t* = −1.14, *P* = 0.2525), time spent outdoors on weekdays (*t* = −1.35, *P* = 0.1781), time spent outdoors on weekends (*t* = −0.36, *P* = 0.7168), and commuting time (*t* = −0.95, *P* = 0.3399).

### Subjective Sleep Characteristics of Participants at Risk of DSWPD

Table 2 outlines the sleep parameters of the participants at risk of DSWPD and the non-DSWPD group. Both bedtime and wake-up time on weekdays were significantly later for the group at risk of DSWPD than for the non-DSWPD group (bedtime: *t* = −6.55, *d* = 0.33, *P* < 0.001; wake-up time: *t* = −6.43, *d* = 0.37, *P* < 0.001). SOL was significantly longer, and sleep duration was shorter in the group at risk of DSWPD (SOL: *t* = −12.49, *d* = 0.59, *P* < 0.001; sleep duration: *t* = 3.84, *d* = 0.20, *P* < 0.001), whereas there was no difference in TIB between the two groups (*t* = −0.28, *d* = 0.01, *P* = 0.7815). Similarly on weekends, the group at risk of DSWPD had a later bedtime and wake-up time, and longer SOL than the non-DSWPD group (bedtime: *t* = −6.43, *d* = 0.38, *P* < 0.001; wake-up time: *t* = −6.11, *d* = 0.34, *P* < 0.001; SOL: *t* = −10.23, *d* = 0.50, *P* < 0.001; sleep duration: *t* = 1.38, *d* = 0.10, *P* = 0.1669; TIB: *t* = −1.46, *d* = 0.11, *P* = 0.1449). SJL was significantly larger in the group at risk of DSWPD (SJL: *t* = −1.99, *d* = 0.13, *P* < 0.05).

**Table 2:** Sleep behaviors of the participants at risk of delayed sleep-wake phase disorder (DSWPD) and those who were not (*n* = 7,810).

	**Non-DSWPD (*****n*** **= 7,473)**		**At risk of DSWPD (*****n*** **= 337)**		***P*** **-Value**	**Effect size (*d*)**
	**Mean h:min**	**SD h:min**	**Mean h:min**	**SD h:min**		
**Weekdays**						
Bedtime	0:10	1:32	0:44	1:50	<0.001	0.33
Sleep onset latency	0:28	0:30	0:50	0:41	<0.001	0.59
Sleep onset	0:38	1:39	1:33	2:01	<0.001	0.58
Wake-up time	7:02	1:37	7:38	2:13	<0.001	0.37
Sleep duration	6:24	1:33	6:04	1:57	<0.001	0.20
Time in Bed	6:53	1:30	6:54	1:56	0.7815	0.01
Mid sleep point	3:50	1:29	4:35	1:53	<0.001	0.53
**Weekends**						
Bedtime	0:42	1:48	1:21	2:16	<0.001	0.38
Sleep onset latency	0:36	0:36	0:56	0:43	<0.001	0.50
Sleep onset	1:18	1:58	2:18	2:25	<0.001	0.50
Wake-up time	9:01	2:01	9:42	2:35	<0.001	0.34
Sleep duration	7:46	1:51	7:37	2:29	0.1669	0.10
Time in Bed	8:21	1:46	8:30	2:20	0.1449	0.11
Mid sleep point	4:38	1:46	5:30	2:17	<0.001	0.49
**Social jetlag**						
Social jetlag	1:21	1:30	1:31	1:48	<0.05	0.13

*SD, standard deviation*.

*Social jetlag = difference between mid-sleep on weekdays as workdays and weekends as free days. P-values and effect sizes were derived from the analysis of the t-test*.

As for students, compared with those without the risk of DSWPD, those at risk of DSWPD had significantly later bedtime and wake-up time with longer SOL and TIB (bedtime: *t* = −4.58, *d* = 0.30, *P* < 0.001; wake-up time: *t* = −6.36, *d* = 0.49, *P* < 0.001; SOL: *t* = −11.91, *d* = 0.78, *P* < 0.001; TIB: *t* = −2.29, *d* = 0.15, *P* < 0.05); however, there was no statistical difference in sleep duration during weekdays between the two groups (*t* = 1.67, *d* = 0.11, *P* = 0.0957; Table 3). Meanwhile, TIB and sleep duration were not statistically different between the two groups on weekends (TIB: *t* = −1.46, *d* = 0.09, *P* = 0.1452; sleep duration: *t* = 1.65, *d* = 0.11, *P* = 0.0988). There was also no statistical difference in the social jet lag parameter between the two groups (SJL: *t* = −0.75, *d* = 0.05, *P* = 0.4505).

**Table 3:** Sleep behaviors of the students and non-students at risk of DSWPD and those who were not.

	**Student**						**Non-student**					
	**Non-DSWPD** **(*****n*** **= 4,250)**		**At risk of DSWPD** **(*****n*** **= 243)**		***P*** **-Value**	**Effect size (*d*)**	**Non-DSWPD** **(*****n*** **= 3,223)**		**At risk of DSWPD** **(*****n*** **= 94)**		***P*** **-Value**	**Effect**
												**size (*d*)**
	**Mean h:min**	**SD h:min**	**Mean h:min**	**SD h:min**			**Mean h:min**	**SD h:min**	**Mean h:min**	**SD h:min**		
**Weekdays**												
Bedtime	0:20	1:24	0:46	1:30	<0.001	0.30	23:56	1:40	0:38	2:32	<0.001	0.41
Sleep onset latency	0:25	0:28	0:48	0:41	<0.001	0.78	0:33	0:32	0:54	0:41	<0.001	0.67
Sleep onset	0:45	1:32	1:34	1:37	<0.001	0.53	0:28	1:48	1:32	2:49	<0.001	0.58
Wake-up time	6:57	1:30	7:35	1:46	<0.001	0.49	7:09	1:46	7:44	3:05	<0.05	0.32
Sleep duration	6:11	1:28	6:01	1:54	0.0957	0.11	6:41	1:35	6:12	2:05	<0.05	0.31
Time in Bed	6:36	1:25	6:49	1:54	<0.05	0.15	7:14	1:33	7:06	2:00	0.3978	0.08
Mid sleep point	3:51	1:21	4:35	1:24	<0.001	0.54	3:49	1:39	4:38	2:46	<0.001	0.38
**Weekends**												
Bedtime	0:53	1:42	1:25	1:51	<0.001	0.31	0:28	1:53	1:11	3:07	<0.001	0.38
Sleep onset latency	0:33	0:36	0:55	0:44	<0.001	0.60	0:39	0:36	1:00	0:41	<0.001	0.59
Sleep onset	1:26	1:53	2:20	2:00	<0.001	0.48	1:07	2:02	2:12	3:15	<0.001	0.51
Wake-up time	9:12	2:00	9:56	2:17	<0.001	0.36	8:46	2:01	9:08	3:10	0.0944	0.17
Sleep duration	7:48	1:51	7:36	2:11	0.0988	0.11	7:44	1:52	7:42	3:09	0.8780	0.02
Time in Bed	8:20	1:46	8:30	2:08	0.1452	0.09	8:22	1:45	8:27	2:49	0.6303	0.05
Mid sleep point	4:43	1:41	5:31	1:45	<0.001	0.47	4:32	1:52	5:26	3:19	<0.001	0.46
**Social jetlag**												
Social jetlag	1:29	1:23	1:33	1:23	0.4505	0.05	1:10	1:37	1:25	2:36	0.1539	0.14

*SD, standard deviation*.

*Social jetlag = difference between the mid-sleep on weekdays as workdays and on weekends as free days. P-values and effect sizes were derived from the analysis of the t-test*.

Among non-student individuals, those at risk of DSWPD showed a later bedtime and wake-up time, longer SOL, and shorter sleep duration compared to the non-DSWPD group on weekdays (bedtime: *t* = −3.96, *d* = 0.41, *P* < 0.001; wake-up time: *t* = −3.07, *d* = 0.23, *P* < 0.05; SOL: *t* = −6.31, *d* = 0.67, *P* < 0.001; sleep duration: *t* = 2.95, *d* = 0.31, *P* < 0.05); however, there was no statistical difference in TIB between those at risk of DSWPD and those who were not (*t* = 0.85, *d* = 0.08, *P* = 0.3978). The wake-up time and sleep duration on weekends in both groups were not statistically different (wake-up time: *t* = −1.67, *d* = 0.17, *P* = 0.0944; sleep duration: *t* = 0.15, *d* = 0.02, *P* = 0.8780).

### The Risk of DSWPD and Sleep Problems

**Figure 1:**
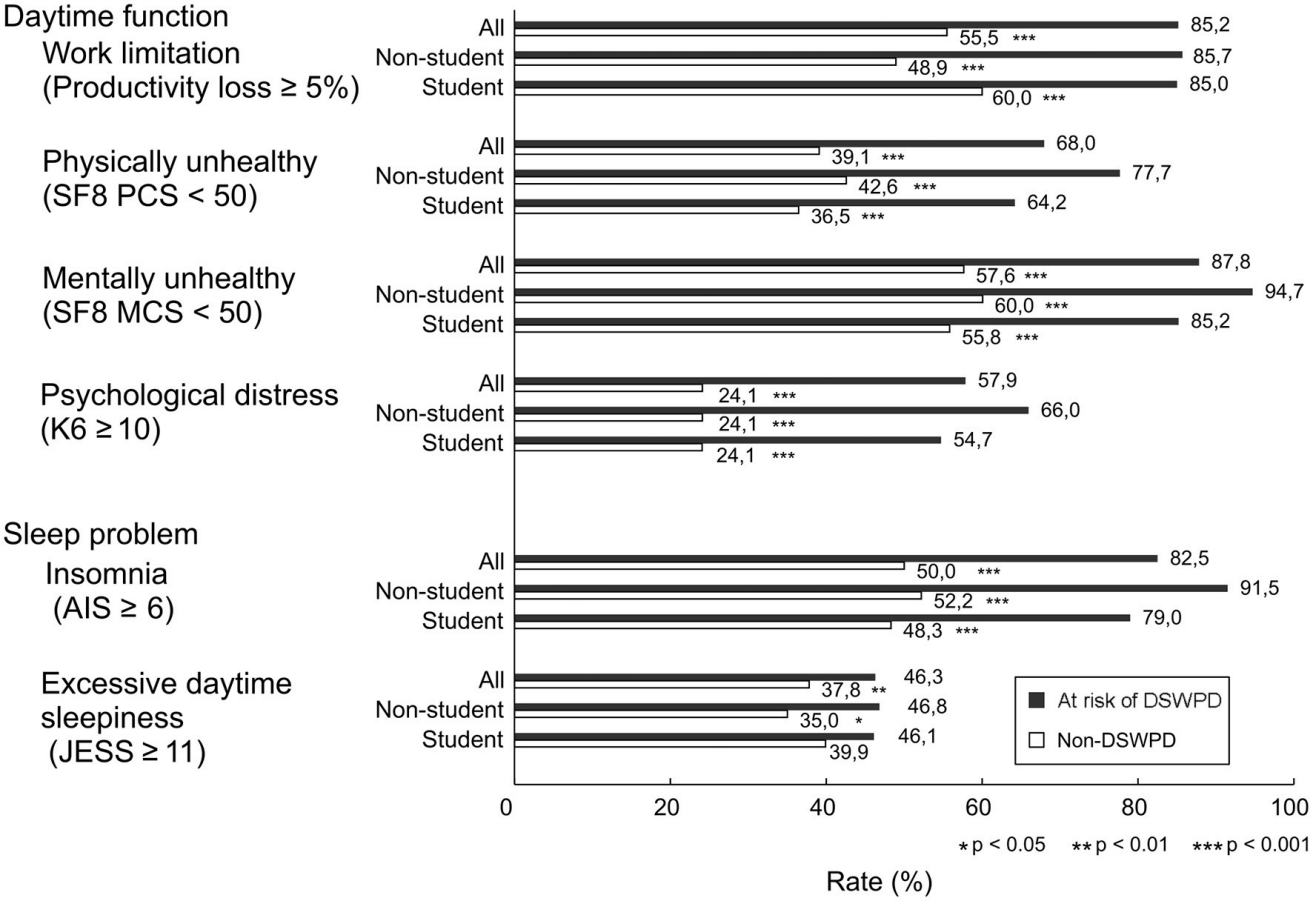
Differences in daytime dysfunction and sleep problem variables of participants at risk of DSWPD and those without DSWPD. Bars represent percentage of people with Productivity loss ≥5%, SF8 PCS <50, SF-8 MCS <50, K6 ≥10, AIS ≥6 and JESS ≥11. Black and white bars indicate the group at risk of DSWPD and the non-DSWPD group, respectively. Significant differences were noted as *P < 0.05, **P < 0.01 and ***P < 0.001.

The percentages of participants with JESS ≥11 and AIS-J ≥6 between those with and without the risk of DSWPD were as follows: JESS ≥11 was 46.3 and 37.8% (χ^2^ = 9.88, ϕ = 0.04, *P* < 0.05), and AIS-J ≥6 was 82.5 and 50.0% (χ^2^ = 136.23, ϕ = 0.13, *P* < 0.05; Figure 1). In the student group, no difference was found in the rate of individuals with ESS ≥11 (46.1% vs. 39.9%, χ^2^ = 3.66, ϕ = 0.03, *P* = 0.056), but the rate of individuals with AIS-J ≥6 was higher in the group at risk of DSWPD (79.0% vs. 48.3% (χ^2^ = 86.56, ϕ = 0.14, *P* < 0.05; Figure 1). For the non-students, similar trends were observed for both sleep problem measures in the comparison between the group at risk of DSWPD and the non-DSWPD group as follows: ESS ≥11 was 46.8 and 35.0% (χ^2^ = 5.58, ϕ = 0.04, *P* < 0.05) and AIS-J ≥6 was 91.5 and 52.2% (χ^2^ = 56.59, ϕ = 0.13, *P* < 0.05; Figure 1).

### The Risk of DSWPD and Daytime Dysfunction

Among the total subjects, the percentage of participants with K6 ≥10, PCS < 50 and MCS <50 in the group at risk of DSWPD and the non-DSWPD group were as follows: K6 ≥10 was 57.9 and 24.1% (χ^2^ = 193.43, ϕ = 0.16, *P* < 0.001), PCS <50 was 68.0 and 39.1% (χ^2^ = 111.64, ϕ = 0.12, *P* < 0.001), and MCS < 50 was 87.8 and 57.6% (χ^2^ = 121.52, ϕ = 0.12, *P* < 0.001), respectively. The percentages of participants with a productivity loss score of ≥5% in the group at risk of DSWPD and the non-DSWPD group were 85.2 and 55.5%, respectively (χ^2^ = 99.37, ϕ = 0.13, *P* < 0.001; Figure 1). In the student group, significant differences in positivity for the worsening of these variables between the group at risk of DSWPD and the non-DSWPD group were also observed as follows: K6 ≥10 was 54.7 and 24.1% (χ^2^ = 113.09, ϕ = 0.16, *P* < 0.001), PCS <50 was 64.2 and 36.5% (χ^2^ = 75.17, ϕ = 0.13, *P* < 0.001), MCS <50 was 85.2 and 55.8% (χ^2^ = 81.11, ϕ = 0.13, *P* < 0.001), and the productivity loss score of ≥5% on the WLQ-SF was 85.0 and 60.0% (χ^2^ = 52.90, ϕ = 0.12, *P* < 0.001; Figure 1). For the non-students, similar trends were observed for all indicators in the comparison between the group at risk of DSWPD and the non-DSWPD group as follows: K6 ≥10 was 66.0 and 24.1% (χ^2^ = 84.65, ϕ = 0.16, *P* < 0.001), PCS <50 was 77.7 and 42.6% (χ^2^ = 45.74, ϕ = 0.12, *P* < 0.001), MCS <50 was 94.7 and 60.0% (χ^2^ = 46.09, ϕ = 0.12, *P* < 0.001), productivity loss score of ≥5% on WLQ-SF was 85.7 and 48.9% (χ^2^ = 40.42, ϕ = 0.13, *P* < 0.001; Figure 1).

### Associated Factors of the Risk of DSWPD

Univariate logistic regression analyses were performed with 10 independent variables (age, length of LCD monitor viewing time, sex, social status, single/married, with/without children, with/without family members in the same household, presence/absence of disease currently treated, presence/absence of the current habit of physical exercise and work, and school hours) to investigate factors associated with the risk of DSWPD. All of these items appeared to be significant in the univariate model. To further examine the factors independently associated with the risk of DSWPD, the above 10 significant variables in the univariate models were included in the multivariate model. The adjusted odds ratios (ORs) and 95% confidence intervals (CIs) of the models are shown in Table 4. The analyses for the entire sample showed that all types of students (high school students, graduate school/university/college students, and other students) were most strongly associated with the risk of DSWPD with full-time employees as the reference (high school students: OR = 3.58, 95% CI = 1.84–6.96, *P* < 0.001; graduate school/university/college students: OR = 2.13, 95% CI = 1.24–3.66, *P* < 0.01; other students: OR = 4.44, 95% CI = 2.32–8.49, *P* < 0.001). Having a disease currently being treated (OR = 3.83, 95% CI = 2.92–5.01, *P* < 0.001), longer LCD viewing time (OR = 1.16, 95% CI = 1.11–1.21, *P* < 0.001), doing habitual exercise (OR = 0.63, 95% CI = 0.48–0.84, *P* < 0.01), and being male (OR = 0.59, 95% CI = 0.42–0.82, *P* < 0.01) also appeared to be significantly associated with the risk of DSWPD (Table 4).

**Table 4:** Logistic regression analyses of factors associated with the risk of DSWPD.

**Variables dichotomized at**	**All**				**Student**				**Non-student**			
	**Univariate relative risk**		**Multivariate relative risk**		**Univariate relative risk**		**Multivariate relative risk**		**Univariate relative risk**		**Multivariate relative risk**	
	**OR**	**95% CI**	**OR**	**95% CI**	**OR**	**95% CI**	**OR**	**95% CI**	**OR**	**95% CI**	**OR**	**95% CI**
**Age**	0.92	0.90–0.95^***^							0.94	0.89–0.99^**^		
**Length of LCD viewing time**	1.17	1.13–1.21^***^	1.16	1.11–1.21^***^	1.16	1.11–1.22^***^	1.15	1.09–1.20^***^	1.20	1.14–1.27^***^	1.18	1.11–1.25^***^
**Sex**												
Female	1.00	Ref.			1.00	Ref.			1.00	Ref.		
Male	0.50	0.36–0.69^***^	0.59	0.42–0.82^**^	0.42	0.29–0.61^***^	0.47	0.33–0.69^***^				
**Social status**												
Full-time employee	1.00	Ref.			–		–		1.00	Ref.		
Part-time worker or inoccupation	2.73	1.76–4.23^***^			–		–		2.73	1.76–4.23^***^	1.88	1.17–3.00^**^
**Homemaker**												
High school student	3.34	2.28–4.89^***^	3.58	1.84–6.96^***^	1.00	Ref.			–		–	
Graduate school/University/College student	2.13	1.44–3.17^***^	2.13	1.24–3.66^**^	0.64	0.48–0.85^**^	0.61	0.44–0.84^**^	–		–	
Other student	4.93	2.99–8.11^***^	4.44	2.32–8.49^***^	1.48	0.98–2.23			–		–	
Single (yes/no)	2.81	1.82–4.35^***^							1.99	1.23–3.22^**^		
Not having children (yes/no)	2.13	1.35–3.36^***^			–				1.86	1.06–3.24^**^		
Living alone (yes/no)	0.69	0.50–0.94^**^			0.65	0.44–0.94^**^						
Having a disease currently being treated (yes/no)	4.32	3.41–5.46^***^	3.83	2.92–5.01^***^	3.74	2.79–5.00^***^	3.54	2.63–4.77^***^	6.55	4.31–9.95^***^	5.60	3.62–8.66^***^
Doing habitual exercise habits (yes/no)	0.77	0.60–0.98^**^	0.63	0.48–0.84^**^	0.59	0.45–0.78^***^	0.58	0.43–0.79^***^				
Working/School hours	0.95	0.90–0.99^**^										

*Cl, confidence interval; OR, odds ratio; LCD, liquid crystal display; ns: not significant*.

^*^ *P < 0.05,*

** *P < 0.01 and*

*** *P < 0.001*.

The adjusted model for students yielded significantly associated factors of the risk of DSWPD as follows: having a disease currently being treated (OR = 3.54, 95% CI = 2.63–4.77, *P* < 0.001), longer monitor viewing time (OR = 1.15, 95% CI = 1.09–1.20, *P* < 0.001), having habitual exercise (OR = 0.58, 95% CI = 0.43–0.79, *P* < 0.001), and being male (OR = 0.47, 95% CI = 0.33–0.69, *P* < 0.001), and showed that the odds of the risk of DSWPD in graduate school/university/college students were lower when referenced with high school students (OR = 0.61, 95% CI = 0.44–0.84, *P* < 0.01; Table 4). Meanwhile, the adjusted model for non-students showed significant factors associated with the risk of DSWPD as follows: having a disease currently treated (OR = 5.60, 95% CI = 3.62–8.66, *P* < 0.001), longer monitor viewing time (OR = 1.18, 95% CI = 1.11–1.25, *P* < 0.001); However, male sex and the presence of habitual exercise did not appear to be significant. Part-time workers or those unemployed showed higher odds compared to full-time employees among non-students (OR = 1.88, 95% CI = 1.17–3.00, *P* < 0.01; Table 4).

## Discussion

In the third edition of the International Classification of Sleep Disorders (ICSD 3^rd^), the diagnosis of DSWPD in clinical settings is made based on a patient's history of chronic or recurrent complaints of symptoms of insomnia with a stable delay in the timing of the major sleep and wake period confirmed with at least 7 days (preferably 14 days) recordings of a sleep diary or actigraphy (1). However, sleep diaries are simple but labor-intensive to record an individuals' sleep-wake status every day. Actigraphy is generally accepted as an objective tool for measuring the sleep-wake schedule, but it is difficult to administer widely, especially to large samples in epidemiological studies. Therefore, a simple and useful assessment tool for the screening of DSWPD is desirable. To date, most epidemiological studies on DSWPD have employed a self-reported questionnaire based on the criteria items of the disorder on the ICSD (9, 12, 13). In this questionnaire, the information regarding the inability to fall asleep at the desired bedtime, which is mandatory for the clinical diagnosis of DSWPD, is lacking (9, 12, 13). Considering this, in the present study, we used the J-BRIAN-SR, which can evaluate sleep and circadian rhythm measures, including difficulty in initiating sleep at the desired bedtime and difficulty waking up, together with a comprehensive evaluation of the severity of depression. Recently we confirmed that a cut-off J-BRIAN-SR score of 40 points could distinguish patients affected with DSWPD from healthy controls with high sensitivity and specificity (16). However, in the present study, considering that the BRIAN score is likely to be higher in younger generation, especially in their twenties (16, 34), we added information on days per month of missing work, school, or daily routines as additional criteria for individuals at risk of DSWPD screening as DSWPD is frequently associated with increased absenteeism and poor performance in work and social life (35).

In the present study, the risk of DSWPD judged with the above criteria in the young Japanese population was 4.3%, which is similar to those in recent studies conducted in Western countries using self-reported questionnaires (9, 12, 13). In addition, the prevalence in women was higher than that in men, which is also in line with previous studies (8, 19). However, some studies have shown that the prevalence in women is not necessarily high (7, 9). Considering that the effect size for the sex distribution between the group at risk of DSWPD and the non-DSWPD group in our results was small (ϕ = 0.05), sex differences in the prevalence of the disorder could be small even if there is a certain difference. DSWPD is generally characterized by a delayed sleep schedule, with subjective sleep problems due to misalignment of the circadian rhythm. In this regard, sleep behaviors on workdays of the participants at risk of DSWPD as a whole showed approximately 35 min later bedtime, 20 mins longer SOL, and 20 min shorter sleep duration, which is consistent with those in the Norwegian study: approximately 25 min later bedtime, 40 min longer SOL, and 20 min shorter sleep duration in students with DSWPD (9). For subjective sleep problems, the proportion of participants who felt daytime sleepiness and insomnia in the group at risk of DSWPD were higher than those in the non-DSWPD group.

The present study comprehensively investigated the impact of the risk of DSWPD on daytime function by targeting work limitations, depression, and physical and mental quality of life. The work limitations in our study were evaluated using WLQ developed by Lerner et al (29). To our knowledge, this is the first attempt to employ the WLQ in a large epidemiological survey of DSWPD. The results revealed that the percentage of participants who felt work limitations was significantly higher in the group at risk of DSWPD, and this trend was seen in both students and non-students. This is in line with an Australian study on the DSWPD high-risk group, which showed increased functional impairment in work, school, and day-to-day operations compared to the low-risk group (35). Previous studies showed that young individuals with DSWPD are likely to experience depression and deterioration of mental health (3, 9, 13, 14, 36). Furthermore, they were considered to be at a higher risk, compared to the general population, of incurring self-destructive behaviors, including self-harm and suicidality (9). In accordance with these, the present study showed trends for worsening of the depression score and the physical and mental quality of life in the group at risk of DSWPD. This suggests that circadian misalignment increases individuals' vulnerability to developing mental dysfunction and that younger individuals at risk of DSWPD should be carefully monitored.

Notably, logistic regression analysis with sociodemographic and lifestyle variables revealed that nocturnal LCD viewing time was positively associated with the risk of DSWPD among all participants. Exposure to a light-emitting device before bedtime is likely to delay the circadian phase of melatonin secretion (37). There is also a report in which some patients with DSWPD demonstrated hypersensitivity to nighttime suppression of melatonin following light exposure (38). Therefore, prolonged exposure to LCD screen-based media, such as TVs, PCs, and smartphones, from the evening to bedtime may be associated with the development or worsening of DSWPD. Our result is consistent with a recent systematic review in which the length of monitor viewing time was adversely associated with sleep health, primarily *via* delayed bedtimes and reduced sleep duration among school-aged youths (39). In contrast, the time of sunlight exposure was not statistically different between the group at risk of DSWPD and the non-DSWPD group. A previous study with small samples of participants aged between 21 and 72 years showed that individuals with DSWPD had significantly less light exposure in the morning compared to controls. In contrast, a total of 24-h levels of light exposure was not statistically different between individuals with DSWPD and controls (40). Nonetheless, children's lack of natural daylight exposure following increased time spent indoors has been recently related to the occurrence of physiological (e.g., sleep) disorders (41, 42). Therefore, while the reason behind our results remains unclear at this stage, further study would be necessary to clarify the relationship between the timing of light exposure and the formation of circadian phase delay in young individuals.

In contrast, current physical exercise habits were negatively associated with the risk of DSWPD, which is in line with previous reports showing that DSWPD was more prevalent among individuals who exercised less (9, 43). Future prospective studies should clarify whether exercise habits prevent the development of DSWPD.

In this study, a significant association between positivity for currently treated disease and the risk of DSWPDs was recognized. Mental disorders such as mood disorders are often comorbid with DSWPD (9, 13, 36). Moreover, disease contraction *per se* and treatment, especially management with hospitalization, may diminish social zeitgeber (44).

Concerning social status, all student categories aged 15 years or older were significantly associated with the risk of DSWPD when referenced with full-time employees. Workers in their 20s are known to have an earlier sleep phase due to regular work schedules than college students of almost the same age (10), suggesting that earlier and tighter time constraints due to work schedules contribute to the formation of regular sleep behaviors among workers.

Interestingly, among the student population in the present study, the OR was lower in college students than in high school students. This is because college students may have more flexible school schedules with less obligation to attend school early in the morning than high school students, resulting in fewer absences from school (45, 46).

Among workers, part-time employees and unemployed individuals had a higher OR for the risk of DSWPD than full-time employees in the present study. For the mechanism of this phenomenon, the following two reasons should be considered: these individuals may not have been able to work on a fixed schedule starting from morning hours due to the effect of DSWPD; conversely, the sleep-wake schedule, especially in part-time employees, was prone to be delayed as a result of a loose work schedule. In addition, a previous study reported results inconsistent with ours (7). Therefore, a future prospective study focusing on the relationship between work status and DSWPD development is required.

This study has several limitations that deserve mentioning. First, we employed a cross-sectional design, which has limitations in revealing the causal relationship between the risk of DSWPD and associated factors. Therefore, longitudinal research on this topic will be necessary in the future. Second, the risk of DSWPD was determined using the self-reported number of days of missing work, school, or daily routines, in addition to J-BRIAN-SR. The J-BRIAN-SR is considered to cover the characteristics of DSWPD comprehensively since the scale may evaluate social dysfunction with the disorder (16). However, young people tend to have a relatively high BRIAN score which may lead to the overestimation of the risk of DSWPD (16, 34). Considering this, we used the self-reported number of non-attendance days additionally. With regard to missing days, we did not collect the data about the reason of missing work, school or daily routines in the present study since it is difficult to distinguish whether non-attendance comes from DSWPD-related symptoms or not with a self-reported questionnaire. A previous study on the prevalence of DSWPD targeting a large number of adolescents used both official register-based and self-reported information to determine non-attendance at school, which did not specify the reason of non-attendance (8). By using this, the study revealed that adolescents with DSWPD had significantly higher odds ratios for non-attendance at school (8). The reason for missing work or school or daily routines in our study could not be identified; however, we believe that the supportive criteria was helpful for the screening of individuals at risk of DSWPD. Nevertheless, clinical interviews and measurements of sleep variables using tools such as sleep diaries or actigraphy were not performed in the present study. In addition, we were unable to calculate the delays in the ideal sleep schedules based on their sleep variables. Accordingly, we were unable to provide a complete definition of DSWPD in the young individuals included in this study. For this reason, the screening-positive cases in this study remain at risk of DSWPD. Finally, the web-based survey may have caused a certain type of sampling bias. In particular, research panels are thought to include a high proportion of people with high accessibility to the Internet (47). Moreover, individuals with a high interest in the content of this survey may be more prone to participate (48, 49). Additionally, in this study, 76.4% of the respondents were female, which was consistent with the trend observed in recent web-based studies (9). This limitation may have contributed to higher prevalence in women than in men.

## Conclusion

This study was the first attempt to use the J-BRIAN-SR, a new validated questionnaire, for the screening of individuals at risk of DSWPD. This study showed that the overall proportion of young Japanese individuals at risk of DSWPD was 4.3%. Individuals at risk of DSWPD were likely to have subjective sleep problems and deteriorated daytime functioning, such as depression and anxiety, presenteeism, and poor health-related QoL. Lack of physical exercise habits and increased length of nighttime LCD viewing time were associated with the risk of DSWPD in this population. In addition, students were more likely to be at risk of DSWPD than workers were, even though they were quite similar ages. Future longitudinal studies are required to investigate the chronicity of the disorder and its associated factors.

## Data Availability Statement

The original contributions presented in the study are included in the article/supplementary material, further inquiries can be directed to the corresponding author.

## Ethics Statement

The studies involving human participants were reviewed and approved by the Ethics Committee of the Neuropsychiatric Research Institute (Tokyo, Japan). Written informed consent to participate in this study was provided by the participants' legal guardian/next of kin. Written informed consent was obtained from the individual(s), and minor(s)' legal guardian/next of kin, for the publication of any potentially identifiable images or data included in this article.

## Author Contributions

All authors listed have made a substantial, direct, and intellectual contribution to the work and approved it for publication.

## Funding

This work was supported by Grants-in-Aid for Scientific Research (grant number 18K07581).

## Conflict of Interest

YI has received speaker's honoraria from Eisai Co., Ltd., Otsuka Pharmaceutical Co., Ltd., Takeda Pharmaceutical Co., Ltd., Astellas Pharma Inc., and MSD K.K. and consultation fee from Eisai Co., Ltd. YK has received research funding from Otsuka Pharmaceutical Co., Ltd. and lecture fee from Takeda Pharmaceutical Co., Ltd. IO has received grants from NEC Solution Innovators and personal fees from Otsuka Pharmaceutical, MSD K.K., and Eisai Co., Ltd. for projects unrelated to the submitted work. The remaining authors declare that the research was conducted in the absence of any commercial or financial relationships that could be construed as a potential conflict of interest. The handling editor declared a past co-authorship with one of the authors YI.

## Publisher's Note

All claims expressed in this article are solely those of the authors and do not necessarily represent those of their affiliated organizations, or those of the publisher, the editors and the reviewers. Any product that may be evaluated in this article, or claim that may be made by its manufacturer, is not guaranteed or endorsed by the publisher.
